# Use of the Xpert Breast Cancer STRAT4 for Biomarker Evaluation in Tissue Processed in a Developing Country

**DOI:** 10.1093/ajcp/aqab016

**Published:** 2021-05-29

**Authors:** Marcellin Mugabe, Kenneth E Ho, Deo Ruhangaza, Dan Milner, Belson Rugwizangoga, Victor C Chu, Natalie C Wu, Annaliza Rizo, Jodi M Weidler, Wendy Wong, Michael Bates, Jane E Brock

**Affiliations:** 1 Windhoek Central Hospital, Windhoek, Namibia; 2 Division of Oncology Research & Development, Cepheid, Sunnyvale, CA, USA; 3 Butaro Cancer Center of Excellence, Butaro, Rwanda; 4 American Society for Clinical Pathology, Chicago, IL, USA; 5 University of Rwanda, School of Medicine and Pharmacy, Kigali, Rwanda; 6 Department of Pathology, Brigham and Women’s Hospital, Boston, MA, USA

**Keywords:** Breast cancer assays, STRAT4, Estrogen receptor, Human epidermal growth factor receptor 2, mRNA, *ESR1*, *ERBB2*, GeneXpert, LMIC

## Abstract

**Objectives:**

Breast cancer immunohistochemistry (IHC) biomarker testing is limited in low-resource settings, and an alternative solution is needed. A point-of-care mRNA STRAT4 breast cancer assay for ESR1, PGR, ERBB2, and MKi67, for use on the GeneXpert platform, has been recently validated on tissues from internationally accredited laboratories, showing excellent concordance with IHC.

**Methods:**

We evaluated STRAT4/IHC ESR1/estrogen receptor (ER), ERBB2/human epidermal growth factor receptor 2 (HER2) concordance rates of 150 breast cancer tissues processed in Rwanda, with undocumented cold ischemic and fixation time.

**Results:**

Assay fail/indeterminate rate was 2.6% for ESR1 and ERBB2. STRAT4 agreement with ER IHC was 92.5% to 93.3% and 97.8% for HER2, for standard (1x) and concentrated (4x) reagent-conserving protocols, respectively. Eleven of 12 discordant ER/ESR1 cases were ESR1- negative/IHC-positive. These had low expression of ER by IHC in mostly very small tumor areas tested (7/12; <25 mm^2^). In two of three discordant HER2 cases, the STRAT4-ERBB2 result correlated with the subsequent fluorescence in situ hybridization (FISH) result. STRAT4-ERBB2 results in 9 of 10 HER2-IHC equivocal cases were concordant with FISH.

**Conclusions:**

The STRAT4 assay is an alternative for providing quality-controlled breast cancer biomarker data in laboratories unable to provide quality and/or cost-efficient IHC services.

**Key Points**

- Obtaining breast cancer biomarkers by immunohistochemistry (IHC) is limited in low-resource settings due to reagent cost, availability, and lack of technical skills, and an alternative solution is needed.
- A near point-of-care messenger RNA STRAT4-*ESR1/ERBB2* breast cancer biomarker assay, for use on the GeneXpert platform, had excellent concordance with estrogen receptor and HER2-IHC in tissue fixed and processed in Rwanda.
- The STRAT4 assay is an excellent alternative for providing quality-controlled breast cancer biomarker data in laboratories unable to provide quality and/or cost-efficient IHC services.

Breast carcinoma ranks as the biggest cancer-related killer of women, with an estimated incidence worldwide of 2 million cases per year and over 600,000 deaths per year, more than half of them in low- and middle-income countries (LMCs).^1^ The health care systems of countries committed to providing cancer care for their people strive to offer complete services that include comprehensive diagnostic services (both imaging and pathology) and therapeutic services that include surgery, radiation therapy, medication, and palliative care. However, the availability of these services is frequently restricted in LMCs through lack of resources, limited infrastructure, and lack of skilled laboratory and health care professionals. Diagnostic workflows that are standard of care in high-resource settings are unrealistic in LMCs, and Rwanda is no exception. Disruptive innovations are required to solve the challenge of providing health care.^2^

Prior to deciding on breast cancer treatment, key information from a diagnostic biopsy procedure is needed: tumor subtype, grade, and prognostic biomarker expression by immunohistochemistry (IHC) for estrogen receptor (ER), progesterone receptor (PR), human epidermal growth factor receptor 2/neu (HER2), and frequently the proliferative marker, Ki-67.^3^ With positive results for ER and/or PR, patients are eligible for an endocrine therapeutic (tamoxifen or aromatase inhibitor) to reduce risk of recurrence by ~50%.^3-5^ With positive HER2 results, HER2-targeted monoclonal antibody therapy is recommended (trastuzumab or biosimilar).^3^ High-risk features, such as high histologic grade, ER-negative or HER2-positive status, and advanced stage, all influence the decision of whether chemotherapy will also improve patient survival (improved by ~30% with receipt of chemotherapy across all grades, stages, and biomarker profiles).^3,6^ Many thousands of cases of breast cancer go undiagnosed and untreated in LMCs. A report of the cancer burden in Malawi between 2007 and 2010 found that fewer than 20% of cancer cases clinically documented were actually confirmed with a diagnostic biopsy specimen due to lack of access to diagnostic services.^7^ Limited and underestimated cancer burden data are typical for LMCs.

IHC services are expensive and technically challenging to provide. For example, in Rwanda, for a population of 12 million, only two hospitals currently perform IHC: Butaro Cancer Center of Excellence (BCCE) and King Faisal Hospital. The BBCE laboratory has historically been staffed by highly skilled technicians trained in the United States to perform IHC, with excellent attention to quality control, and there is a highly skilled pathologist (D.R.) reading the assays. Unfortunately, this is difficult to sustain and not the norm for low-resource settings, and quality control is an immense challenge in IHC laboratories in all LMCs. Even with centralized testing and ER being the most frequent IHC assay at BCCE (as it is in all pathology laboratories in LMCs), it is still a challenge to ensure year-round provision of all IHC reagents and to handle budget constraints. Reagent use must be efficient, and even with good management, basic laboratory stocks are frequently insufficient to meet a country’s clinical needs. BCCE is currently the nation’s only cancer treatment center, and it regularly suffers from stock-outs of key reagents for performing IHC.

An innovative alternative solution that bypasses IHC for providing biomarker status is to use the Xpert Breast Cancer STRAT4 (CE-IVD) assay (Cepheid). It makes semiquantitative measurements of four breast biomarkers, *ESR1*, *PGR1*, *ERBB2*, and *MKi67* messenger RNAs (mRNAs), and one reference gene (*CYFIP1*) using whole sections cut from formalin-fixed, paraffin-embedded (FFPE) blocks in ~75 minutes on an automated diagnostic platform, the GeneXpert (GX) (Cepheid).^8,9^ After brief sample preparation using FFPE lysis reagents, the STRAT4 cartridge extracts nucleic acids, amplifies them, and detects them using reverse transcription polymerase chain reaction (RT-PCR) on the GX platform (multiplexed automated PCR). Comparisons of IHC vs mRNA in breast cancer biomarkers are not novel, and mRNA evaluation of breast biomarkers is both validated in clinical trials and a routine reported component of the breast cancer Recurrence Score assay from Genomic Health.^5,10,11^ What is innovative and completely novel about this study is that the STRAT4 assay is an all-in-one breast cancer assay for use on the widely available GeneXpert platform. It is a near point-of-care test that can be performed in a laboratory setting, with all required reagents included either in the FFPE lysis package or within the cartridge itself, including quality controls, and results are automatically reported out as positive, negative, or assay fail. Hence, this assay is of interest in the global breast cancer diagnostics market, where more than half of breast cancer occurs.

The STRAT4 assay is a simple to perform molecular test in a basic laboratory setting, and over 25,000 GX machines are currently in use in 182 countries, including 75 currently in use in laboratories and facilities across Rwanda. The GX machine is already in many more hospitals in LMCs than have IHC capability due to the popularity of a rapid GX tuberculosis assay (MTB/RIF assay). The STRAT4 assay has been optimized and validated using over 500 clinical samples, and it has a high concordance (overall percent agreement [OPA]) with conventional immunohistochemistry breast cancer biomarkers in whole FFPE sections tested, in the region of 97.8% for *ESR1* (compared with ER-IHC) and 93.3% for *ERBB2* (compared with HER2-IHC/fluorescence in situ hybridization [FISH]).^8,9^ This assay obviates the need to procure multiple different reagents, has internal quality controls built into the assay that minimize skills needed by technical staff, and minimizes skills needed by pathologists evaluating the breast cancer biomarker data. There is no other assay previously reported or currently commercially available that can perform this task.

The goal of this study was to test the STRAT4 assay with biopsy and excision samples routinely obtained, fixed, and processed in Rwanda and compare STRAT4 results with high-quality IHC/FISH testing performed in a US-based academic reference laboratory. The IHC testing was performed in the United States as part of a nonprofit Partners in Health program for Rwanda prior to IHC testing coming on site at BCCE. Clinicians and pathology laboratory staff at BCCE in Rwanda are trained in good tissue handling, but even so, we surmised that the published excellent STRAT4 data may be challenging to replicate in samples that may be suboptimally handled and where cold ischemic time (CIT) and fixation time are not documented. We compared STRAT4-*ESR1* and *ERBB2* results concordance with IHC ER and HER2 results, as well as HER2 FISH results where HER2-IHC results were equivocal (2+) or discordant with ERBB2. Finally, with efficient use of reagents in mind, we evaluated whether reducing the volume of reagents consumed per case prepared negatively affected results in routinely processed specimens.

## Materials and Methods

Institutional review board approval was obtained from both Brigham and Women’s Hospital (BWH) and the Rwanda National Ethics Committee for the study. A total of 150 consecutive invasive breast tumor samples obtained between 2012 and 2014, including 91 core biopsy specimens and 51 excision specimens in FFPE blocks, were used for this study, with most samples originating at Butaro Hospital. FFPE blocks were subsequently shipped to BWH in Boston, Massachusetts, for histopathologic confirmation of diagnosis and biomarker testing. Histopathology slide evaluation, ER (SP1 clone), and HER2-IHC (SP3 clone) assays were performed at BWH. STRAT4 mRNA assays were performed at Cepheid following the manufacturer’s instructions for use, and results were reported to BWH. All STRAT4 test results generated for this study were considered for research use only and were not used for making patient care decisions. HER2 FISH was performed by NeoGenomics. An additional ER-IHC (6F11 clone) was performed at the Keck School of Medicine.

### IHC Protocol

FFPE sections measuring 4 µm were immunostained according to manufacturer recommendations using the EnVision+ System-HRP (DAKO). Primary antibodies included ER-SP1 (dilution 1:200; Lab Vision) and HER2-SP3 (dilution 1:100; Lab Vision). A DAKO polymer secondary antibody system was used (Envision Poly K4011). Slides were scored for ER positivity and HER2 positivity according to American Society of Clinical Oncology (ASCO)/College of American Pathologists (CAP) guidelines.^12,13^ Discrepant cases were retested using an alternative ER antibody clone (6F11) and an independent pathology evaluation (Mike Press, Keck School of Medicine). PR-IHC and Ki-67 IHC were not performed due to resource constraints.

### HER2 FISH

All STRAT4-*ERBB2* HER2-IHC–discordant cases and HER2-IHC–equivocal cases (2+) were subsequently analyzed by FISH. FISH was scored according to ASCO/CAP guidelines.^13^ FISH was performed using the Pathvysion HER-2 DNA probe kit (Abbott Laboratories).

### Histopathology Evaluation

Tumor subtype and modified Bloom-Richardson grade (Nottingham grade) were recorded. The area of tumor in a single 4-µm FFPE H&E-stained section was estimated for each sample tested as large (≥51 mm^2^), small (>25-50 mm^2^), or very small (≤25 mm^2^). Tumor cellularity was estimated as the proportion of space in the tumor bed area that the tumor cells occupy (tumor density or sparsity, not relative DNA content of tumor compared with nontumor cells).

### STRAT4 Assay

All tumor samples were tested using both the standard (“1×”) and (“4×”) concentrated lysate methods. Whole tissue sections were used for each method. For each method, a single 4-µm FFPE section was placed into a 1.5-mL tube as a scroll or scraped with a scalpel blade from an unstained section placed on a glass microscope slide. For each 1× standard lysate, 1.2 mL FFPE lysis reagent and 20 µL proteinase K were added to the sample. The sample was briefly vortexed, pulse spun, and then incubated at 80°C for 30 minutes. The lysed sample was transferred to a 5-mL vial, and 1.2 mL 95% or more ethanol was added, and the sample was vortexed again. For the 4× method, a smaller reagent volume was used: 260 µL FFPE lysis reagent, 5 µL proteinase K, and 260 µL 95% or more ethanol.

Following each lysate preparation method, a 520-µL aliquot of the FFPE lysate was then added to a STRAT4 cartridge, and the cartridge was placed in a GeneXpert module. Each single-use, disposable STRAT4 multiplex cartridge is preloaded with all necessary RT-PCR assay reagents, including wash buffers, elution buffers, and lyophilized beads containing primers, enzymes, and nucleotides, including primers and probes for quantitative real-time PCR measurement of the four breast cancer mRNA markers (*ESR1/PGR1/ERBB2/MKi67*) and one reference gene (*CYFIP1*). The test cartridge integrates sample purification, nucleic acid amplification, and detection of the target sequence using real-time RT-PCR and real-time PCR assays. The mRNA assay takes about 75 minutes to amplify a portion of the *ESR1*, *PGR1*, *ERBB2*, and *MKi67* mRNAs and *CYFIP1* reference mRNA within the sample and to generate the test results. The GX system consists of the GeneXpert instrument, barcode reader, a computer, and preloaded software for running tests and viewing the results.

For each tumor sample, cycle threshold (Ct) values for each target gene of interest were obtained, along with simultaneously measured Ct values for the reference gene (*CYFIP*) and an internal control gene (*CIC*). The delta Ct value for each target is calculated by *CYFIP* Ct – target gene and is positive, negative, or indeterminate for expression of *ESR1*, *PGR1*, *ERBB2*, and *MKi67*, based on validated assay cutoffs and sample adequacy specifications. Assays are reported out as positive, negative, or invalid/indeterminate. Final positive or negative results for each marker were then analyzed for concordance (OPA, positive percent agreement, and negative percent agreement) vs IHC and/or FISH measurements for the same markers.

## Results

### **1**× **vs 4**× **Concentrated Lysate**

The standard (1×) vs concentrated (4×) reagent lysate protocols provided valid assay results in 94% to 97% and 97% to 99% of cases across each of the assay targets, respectively. Agreement between the 1× and 4× lysates was 98.0% for *ESR1*, 97.2% for *PGR1*, 98.0% for *ERBB2*, and 95.8% for *MKi67*. The invalid/indeterminate rate with the standard 1× vs concentrated 4× protocols was 2.6% vs 0.7% for *ESR1*, 7.9% vs 4.0% for *PGR1*, 2.6% vs 0.7% for *ERBB2*, and 5.3% vs 2.0% for *MKi67*.

### Estrogen Receptor

**Figure 1:**
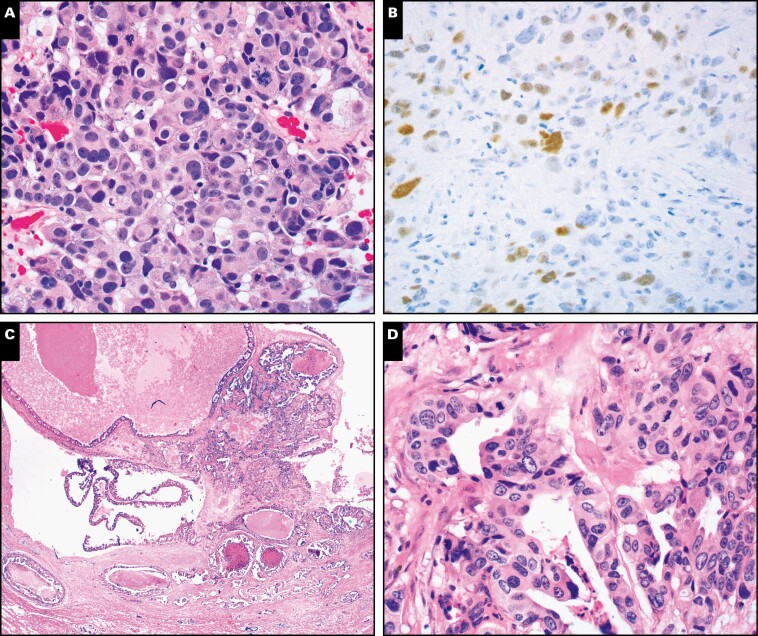

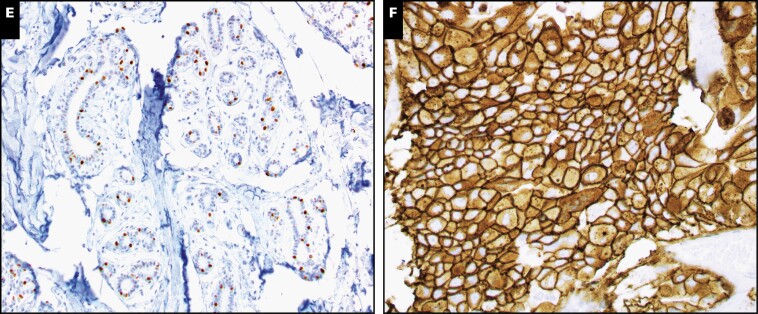
STRAT4-*ESR1* vs ER-IHC discordant cases. **A**, STRAT4-*ESR1*–negative, ER-IHC positive example (case 4 from Table 2). H&E stain. **B**, Case 4 from Table 2. ER (SP1 clone) immunostain shows weak to moderate positive staining in 5% of tumor cells (95% of the tumor was ER negative). **C-F**, STRAT4-*ESR1*–positive, ER-IHC–negative example (case 1 from Table 2). **C**,**D**, Low- and high-magnification H&E show papillary and cystic architecture to this carcinoma. **E**, The tumor was ER negative (figure not shown), and there was extensive normal breast epithelium around the tumor staining positive (shown). **F**, HER2-IHC. STRAT4-*ERBB2* and HER2-IHC were concordant and positive. ER, estrogen receptor; IHC, immunohistochemistry. (**A**, **B**, **D**, **F**, ×200; **C**, **E**, ×100)

Overall STRAT4-*ESR1* agreement with ER-IHC was 92.5% with the 1× lysate and 93.3% with the 4× method **Table 1**. Eleven cases were discordant with the 1× lysate, and 10 cases were discordant with the 4× lysate (12 discordant cases total) **Table 2**. Discordance when testing long-term stored (>2 years) 4-μm sections was resolved in several additional initially discrepant cases by using a fresh cut from the FFPE block. Ten of 11 cases were STRAT4-*ESR1* negative/ER-IHC positive, with ER-IHC showing a range of percentage staining from 1% to 90% and weak to moderate staining ****Figure 1A** and **Figure 1B**** (Table 2). Repeat ER-IHC using the 6F11 clone confirmed the original ER-IHC result using SP1 in five of six discrepant cases. For six other discrepant cases, invasive tumor areas were exceptionally small, and no invasive tumor remained in the tissue block for repeat ER-IHC evaluation with 6F11. The STRAT4-*ESR1*–positive/ER-IHC–negative case had normal breast tissue showing positive internal control staining for ER on IHC and was concordant for *ERBB2*/HER2 results ****Figure 1C**, **Figure 1D**, **Figure 1E**, and **Figure 1F****.

**Table 1:** Standard 1× Lysate and 4× Lysate Protocol STRAT4-*ESR1* vs ER-IHC Results^a^

	STRAT4-*ESR1*, No.					
	1× Lysate			4× Lysate		
ER-IHC	Positive	Negative	Total	Positive	Negative	Total
Positive	65	10	75	68	10	78
Negative	1	71	72	0	72	72
Total	66	81	147	68	82	150

ER, estrogen receptor; IHC, immunohistochemistry.

^a^For the 1× lysate, the overall percent agreement (OPA) was 92.5%, positive percent agreement (PPA) was 86.7%, and negative percent agreement (NPA) was 98.6%. For the 4× lysate, the OPA was 93.3%, PPA was 87.18%, and NPA was 100%.

**Table 2:** Discordant STRAT4-*ESR1* vs ER-IHC Cases^a^

Case No.	STRAT4-*ESR1*	Discordant Lysate	ER-SP1 IHC	ER-6F11 IHC	Tumor Area, mm^2^	Tumor Cellularity, %
1	Positive	1×	Negative (0%)	Negative (0%)	140	40
2	Negative	1× 4×	Positive (60% weak)	Positive (8% weak)	43	80
3	Negative	1× 4×	Positive (20% moderate)	Positive (20% weak)	39	40
4	Negative	1× 4×	Positive (5% moderate)	Positive (14% weak)	24	55
5	Negative	1× 4×	Positive (50% weak)	Positive (2% weak)	28	80
6	Negative	1×	Positive (90% moderate)	—	1	70
7	Negative	1× 4×	Positive (50% weak)	—	7	60
8	Negative	1× 4×	Positive (1%-5% weak)	—	20	70
9	Negative	1× 4×	Positive (30% weak)	—	3	40
10	Negative	1× 4×	Positive (20% weak)	Negative (<1%)	144	30
11	Negative	1× 4×	Positive (30% weak)	—	3	80
12	Negative	4×	Positive (70% weak)	—	2	80

IHC, immunohistochemistry; 1×, standard reagent lysate; 4×, concentrated reagent lysate.

^a^Ten of 12 discordant cases were retested using a different antibody clone (6F11) and reviewed by an independent pathologist (Mike Press, Keck School of Medicine). No invasive carcinoma remained in six cases for retesting (cases 6, 7, 8, 9, 11, 12). Poor tissue preservation was noted in 1 of 12 discrepant cases sufficient to impair histologic evaluation (case 6). All ERBB2/human epidermal growth factor receptor 2 (HER2) results were concordant in the discordant estrogen receptor cases, including five positive HER2 results (cases 1, 5, 9, 10, 11). Tumor area and cellularity are reported for the discrepant cases.

### HER2

**Figure 2:**
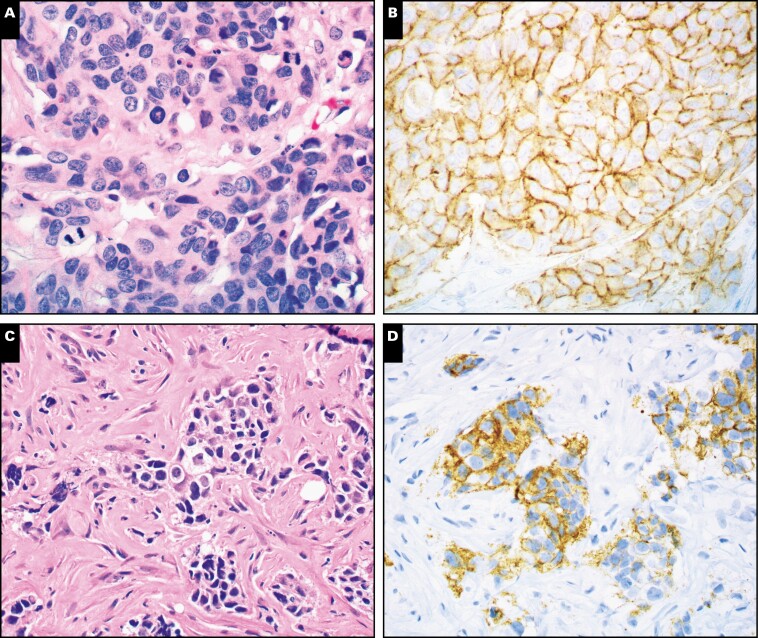
STRAT4-*ERBB2* vs HER2-IHC discordant cases. **A**, HER2-IHC false-positive case, H&E (case 1 in Table 4). **B**, HER2-IHC originally classified as HER2 positive (3+), but this was STRAT4-*ERBB2* negative and negative on HER2 FISH. HER2 IHC shows granular complete membranous staining, rather than crisp complete membranous staining (compare with Figure 1F). In retrospect, this is better classified as equivocal (2+). **C**, HER2-IHC false-negative case, H&E (case 2 in Table 4). **D**, HER2-IHC originally classified as HER2 negative (1+) was positive by STRAT4 and by HER2 FISH. It shows granular incomplete membranous staining. The lack of complete membranous staining may have been due to poor tissue preservation. FISH, fluorescence in situ hybridization; HER2, human epidermal growth factor receptor 2; IHC, immunohistochemistry. (**A-D**, ×200)

STRAT4-*ERBB2* agreement with HER2-IHC was 97.8% with the 1× lysate and 97.8% with the 4× lysate **Table 3**. Three cases (3/150) were discordant, which on subsequent FISH testing identified two results concordant with STRAT4-*ERBB2* results and one concordant with IHC results **Table 4**. The IHC false-positive case showed very granular moderate to strong membranous staining, rather than chicken-wire strong and crisp membranous staining ****Figure 2A** and **Figure 2B****. The IHC false-negative case showed areas of granular partial moderate membranous but no complete membranous staining ****Figure 2C** and **Figure 2D****. STRAT4-*ERBB2* results in equivocal HER2-IHC cases were compared with FISH. Nine of 10 cases had concordant results. The discordant result, STRAT4-*ERBB2* negative/FISH positive, had FISH HER2 copy number in the equivocal range (5.1) and was a low-amplified case (HER2/cep17 ratio 2.32).

**Table 3:** Standard 1× Lysate and 4× Lysate Protocol STRAT4-*ERBB2* vs HER2-IHC Results^a^

	STRAT4-*ERBB2*, No.					
	1× Lysate			4× Lysate		
HER2-IHC	Positive	Negative	Total	Positive	Negative	Total
Positive (3+)	33	1	34	33	1	34
Negative (0, 1+)	2	101	103	2	104	106
Total	35	102	137	35	105	140

HER2, human epidermal growth factor receptor 2; IHC, immunohistochemistry.

^a^For the 1× lysate, the overall percent agreement (OPA) was 97.8%, positive percent agreement (PPA) was 97.0%, and negative percent agreement (NPA) was 99.0%. For the 4× lysate, the OPA was 97.8%, PPA was 97.0%, and NPA was 99.0%.

**Table 4:** Discordant STRAT4-*ERRB2* vs IHC-HER2 Cases^a^

Case No.	STRAT4-*ERBB2*	IHC-HER2	HER2 FISH (HER2/Cep17 = Ratio)
1	Negative	Positive (3+)	Negative (3.05/3.1 = 0.98)
2	Positive	Negative (1+)	Positive (9.15/1.65 = 5.55
3	Positive	Negative (1+)	Negative (3.20/2.90 = 1.1)

FISH, fluorescence in situ hybridization; HER2, human epidermal growth factor receptor 2; IHC, immunohistochemistry.

^a^Two STRAT4 results were concordant with FISH, and one IHC result was concordant with FISH. The ratio reports the average HER2 copy number compared with centromere Cep17 copy number. A ratio of 2.0 or more is a positive FISH result.

### Tumor Area, Subtype, and Cellularity

One-third of cases (51/150) had very small areas of invasive tumor for evaluation (≤25 mm^2^), and 7 of 12 ER-*ESR1* discordant cases seen were in these very small tumors tested (Table 2) **Table 5**. Two of three HER2-*ERBB3*–discordant cases were very small tumor areas (cases 2 and 3 of Tables 4 and 5). There was no difference between the 1× or 4× lysate protocol with respect to tumor area and discordance (Table 5). Sparsely cellular tumors (<50% of tumor bed area occupied by tumor cells) were more prevalent in lobular (two of four cases) and mucinous (two of two cases) subtypes, although most (~90%) were ductal not otherwise specified. Tumor cellularity ranged from 25% to more than 90%, and 21.3% (32/150) had lower cellularity (≤50%). Low cellularity independent of a small tumor area was not notable in discrepant cases (Tables 2 and 5). Lobular, mucinous, and micropapillary subtypes were concordant between STRAT4 and IHC.

**Table 5:** Discordant STRAT4-*ESR1* vs IHC-ER Cases and STRAT4-*ERBB2* vs IHC-HER2 Cases by Tumor Area and Cellularity^a^

	Tumor Area, No.			Tumor Cellularity, No.	
Characteristic	Very Small (≤25 mm^2^)	Small (26-≤50 mm^2^)	Large (≥51 mm^2^)	Low (≤50%)	Normal (≥51%)
Cases	51	43	56	32	118
ER-IHC STRAT4-*ESR1* 1× discordant	6 (of 12)	3	2	4	8
ER-IHC STRAT4-*ESR1* 4× discordant	6 (of 12)	3	1	4	8
Discordant HER2-IHC STRAT4-*ERBB2*	2 (of 3)	0	1	1	2

^a^Approximately one-third of tumors fell in each tumor area category, but discrepant cases were disproportionately in the very small tumor area samples. Low tumor cellularity defined as 50% or less of tumor area occupied by tumor cells was not a notable issue in discrepant cases.

## Discussion

STRAT4 is a near point-of-care test that can be performed in a laboratory setting, with all required reagents included either in the FFPE lysis package or within the cartridge itself, including quality controls, and results are automatically reported out as positive, negative, or assay fail. The novelty of the data presented here is its delivery system: a point-of-care test that can deliver near-patient testing and fast results. This study is also uniquely relevant in that it specifically looks at its use in tissue obtained, fixed, and processed in a low-resource setting, where the assay is intended to be deployed, but compares the assay’s performance with the highest quality assay results obtained in a US academic institution rather than an immunohistochemistry laboratory in a low-resource setting.

We have found excellent overall concordance between a point-of-care mRNA assay, STRAT4, and immunohistochemistry for breast cancer biomarker evaluation for ER and HER2 for 150 samples fixed and processed in Rwanda. Overall STRAT4 agreement with ER-IHC was 92.5% to 93.3% and 97.8% for HER2, using standard volume (1×) and concentrated (4×) reagent volume protocols, respectively. In published and unpublished studies from academic institutions in the United States and Europe, the assay is highly concordant with ER and HER2 (personal communication, Michael Bates, 2020).^8,9^ Using a smaller volume of lysis reagent and ethanol did not compromise results, thus conserving potentially hard-to-obtain supplies. Discordant ER/*ESR1* cases were predominantly STRAT4-*ESR1* negative/IHC positive (11/12 cases) with weak and/or low expression of ER by IHC in mostly very small tumor areas tested (<25 mm^2^). In two of three discordant HER2 cases, the STRAT4-*ERBB2* result correlated with the subsequent FISH result. In 10 cases reported as equivocal by HER2-IHC, STRAT4-*ERBB2* results were concordant with FISH in nine cases. These were archival materials tested, and it was important to use a fresh cut off an FFPE block for optimal STRAT4 testing, as would be routine when testing new diagnoses of breast cancer.

Controlling tissue CIT—the time from removal from patient to sectioning and placing in 10% neutral buffered formalin fixative—and quality of tissue fixation is a major challenge in pathology laboratories. CAP guidelines recommend a CIT of under an hour and fixation between 6 and 72 hours.^12,13^ Particularly in resource-constrained situations, through lack of personnel, resources, and inexperience, tissue is commonly left at ambient temperature for many hours prior to fixation, in the operating room, during transport to the pathology laboratory, and in the pathology laboratory. Prolonged CIT and inadequate tissue fixation result in marked tissue autolysis, impaired tumor subtyping and grading, and challenging biomarker evaluation.^14^

In high-income settings, CAP laboratory standards are typically both strictly adhered to and documented for CAP accreditation of a laboratory. Neither CIT nor fixation time was quantified in our cohort, and despite some morphologic evidence of tissue ischemia (sloughing of epithelium and tissue autolysis) in a number of cases and very small tumor areas tested, the assay fail/indeterminate rate was less than expected, 2.6% (4/150).

Our ER-IHC–negative rate was 48%, STRAT4-*ESR1*–negative rate was 55%, HER2-IHC–positive rate was 23%, and STRAT4-*ERBB2*–positive rate was 25%. These statistics complement data in a recent molecular analysis of breast tumors in a Nigerian population, in whom 68% of tumors tested were ER negative, 25% were HER2 positive, and ER-positive tumors were biologically more aggressive.^15^ In the United States, subtype prevalence is notably different (33% vs ~20% ER negative; 17% vs ~15% HER2 positive for black and white, respectively).^16^ This significant difference in subtype prevalence and aggressiveness of cancer in African populations illustrates the need to know biomarker status for treatment. LMCs across Africa frequently resort to universal treatment of all women with endocrine therapy when biomarker testing is unavailable, but this therapy is likely beneficial in just half of the women and may be harmful in those treated who are ER negative.

Core needle biopsy specimens can contain very small areas of tumor for evaluation. A typical core measures 10 to 20 mm in length by 1.6 to 2.0 mm in diameter (14-12 gauge), and although at least five core samples are recommended (obtaining >100 mm^2^ of tissue), this may not be feasible in low-resource settings where vacuum-assisted biopsies and biopsies requiring just a single insertion of the needle to obtain multiple cores are rarely performed due their extreme expense. Instead, the needle needs to be reinserted into the breast lesion for each core obtained, causing significant patient discomfort. Sixty percent of the samples in our study were core biopsy specimens, and we found one-third of our tumor samples were 25 mm^2^ or less. Half of the discordant cases were seen in smaller tumor area biopsy specimens (1, 2, 3, 3, 7, and 22 mm^2^ original invasive tumor areas, respectively). This exceptionally small invasive tumor area to evaluate may have contributed to the discrepancy in these cases, and pathologists using STRAT4 for biomarker evaluation should be aware that very small tumor areas could provide a false-negative result.

However, the possibility that the negative STRAT4-*ESR1* mRNA result is more clinically useful than a weakly staining positive ER-IHC result must be recognized, too. *ESR1* mRNA levels correlate linearly with risk of recurrence, whereas ER-IHC does not. Data from a European cohort show that ER-IHC assays may still be positive when corresponding *ESR1* mRNA levels are below a positive threshold that more accurately predicts for distant recurrence.^5^ These data suggest that mRNA analyses are the better assay for choosing those who will benefit from treatment. This has been acknowledged by pathologists in the breast cancer field, but there is currently no motivation to exchange ER-IHC assays in high-resource settings for *ESR1* mRNA.

HER2-targeted therapy cost is far beyond the means of most LMC government-subsidized health care systems. Cost reduction solutions are to use biosimilars and reduce therapy duration. The high HER2-positive disease prevalence in our cohort (25% positive, of which 76% is ER negative) and African populations overall must be emphasized and recognized globally, as HER2-targeted therapy offers a high chance of cure. Sixty-five percent of ER-negative and 42% of ER-positive HER2-positive patients can have a pathologic complete response (no residual tumor left) after HER2-targeted therapy alone, and 90% show response.^17^

When HER2-targeted therapies are available, the STRAT4 assay has the advantage of eliminating the need for expensive and technically challenging FISH in the cohort of HER2-IHC–equivocal cases. HER2-IHC–equivocal rates can be anywhere from 8% to 25% of cases tested in the United States depending on the HER2 clone and assay used and could be predictably higher in LMCs, with higher prevalence, the challenges of assay quality control, and limited professional experience reading assays.^18^ In Rwanda, those currently testing equivocal for HER2 are considered negative, as FISH is not available, and 30% of these patients could have benefited from treatment.

In 2020, Rwanda reported a breast cancer annual incidence of 29.1 per 100,000 population, corresponding to 1,237 new cases.^19^ However, biomarker studies are performed on fewer than 25% of these cases in Rwanda’s hospitals (~250 per annum). This mismatch is not because 75% of Rwanda’s women with breast cancer have their biomarker analysis performed out of country in a private laboratory but because many women do not access a breast cancer biomarker diagnostic assay. This can be for a multitude of reasons such as unwillingness/inability to seek care, unwillingness/inability to travel to a facility capable of procuring a diagnostic biopsy, inability to afford the diagnostic assay (despite income-based sliding-scale subsidies), and patient or doctor perception that the diagnostic assay will not alter treatment and outcome in the palliative setting. This surprising statistic that less than 25% of women with breast cancer get biomarker evaluation serves as an indicator of undocumented unmet needs in Rwanda and can easily be extrapolated to other low-income countries that have nothing close to the health care coverage Rwanda provides its people. Despite Rwanda admirably spending 6.5% of its gross domestic product (GDP) on health care and with near-universal health insurance coverage, diagnostic services and therapeutic services are still inadequate to fully serve its population and need to be expanded over time.

A cost-benefit analysis puts IHC reagent costs per case at approximately US$60 based on economic models for seven sites in Africa (personal communication, Dan Milner, 2020). This is approximately the same price for both an automated platform and a manual IHC and does not take into account laboratory equipment setup costs. Sixty dollars is relatively astronomical in a low-income country like Rwanda, where per capita GDP is $772.94 (2019 World Bank data). Although HER2 FISH or chromogenic in situ hybridization is not routinely performed in Rwanda for IHC-equivocal cases, if it were, this would double the cost of the assay for the 7% of breast cancer cases that tested HER2 equivocal in our study. In settings where FISH is performed on equivocal cases, this clearly tips the scales in favor of using the STRAT4 assay when the cartridge price point is comparable to IHC cost alone.

The price has not yet been set for the STRAT4 cartridge in African countries, but Cepheid has a strong track record of providing affordable assays in the developing world (the MTB/RIF cartridge in eligible low-income countries is significantly subsidized at ~$10/cartridge). The goal in breast cancer biomarker evaluation is a real sustainable solution that is more affordable and easier to quality control than IHC, to allow expansion of high-quality testing. Considerations for the cost-benefit analysis include the following:

1. Cost of setting up IHC capacity ($10,000-15,000 for manual IHC and $30,000-50,000 for automated IHC, excluding validation of individual assays), compared with a GeneXpert device (the omni, a 1-module device, is <$5,000; 2-module GX, ~$12,000; 4-module GX, ~$17,000; and 16-module GC, ~$70,000; excluding validation of individual assays).
2. Reliability of stock supply: one self-contained cartridge vs many reagents needed for IHC, each of which can be challenging to keep stocked. Vendor availability is also a significant factor.
3. Shelf life of stock (similar, in the order of 1-2 years for IHC reagents and GeneXpert cartridges).
4. Reliability of GeneXpert and access to maintenance of devices. The GeneXpert can be remotely accessed by Cepheid to troubleshoot issues with devices, and servicing is widely available on all continents given the prevalence of the devices and their widespread routine use.
5. Proximity of testing to patient location. Current estimates from Cepheid report approximately 75 GX devices in Rwanda, 250 in Uganda, 300 in Ethiopia, 250 in Kenya, 150 in Ghana, 400 in South Africa, 250 in Tanzania, 400 in Nigeria, and 130 in Malawi, to give some sense of the widespread distribution of these devices. Although these units are predominantly used for TB assays and many may not be available for STRAT4 assay use due to high-volume TB testing, the number of potential test sites far outnumbers the sites capable of performing breast cancer biomarker evaluation by IHC in every country.
6. Turnaround time for the assays (<2 hours for STRAT4 vs 8 hours for IHC).
7. Quality controls integrated into the STRAT4 assay vs requiring human technical skill to ensure all quality controls are adhered to during the IHC workflow and when interpreting the results. Critical steps that need to be controlled for IHC include antigen retrieval; preparation of antibodies including validation studies for each batch; preparation of reagents; incubation, washing, and counterstaining; and use of both positive and negative controls.
8. Retest costs in the event of a failed assay (2.6% for STRAT4 in our study). The fail rate for IHC is very much dependent on laboratory quality controls in place.
9. Increased capacity to test close to the patient, as a near point-of-care test, outside of the setting of laboratories accredited to perform IHC. In Rwanda, this could be in the 42 district hospitals that have GeneXperts and that are staffed by physicians who could potentially perform a biopsy of a palpable mass (incisional biopsies, core biopsies, or a very simple fine-needle aspirate). This would significantly expand and speed up testing in Rwanda.
10. Additional utility of having *PGR* and MKi67 proliferation data for treatment decision making (not currently performed due to cost constraints).

A cost-benefit validation study on site in Rwanda is currently under way, and there are multiple similar validation studies ongoing across Africa, amply demonstrating its perceived worth to those who need innovative solutions to the challenge of breast cancer biomarker diagnostics. Several US and European validation studies, including an external quality assurance study, will shortly be published using this assay (personal communication, Michael Bates, 2021), adding to the data regarding the quality of this assay, and increasing the confidence of those who need it to use it. As data accumulate, there may even be some calls across the United States and Europe to use this assay, given the rapid turnaround time, internal quality controls provided, more clinically relevant *ESR1* result, and noninferiority for *ERBB2* vs HER2-IHC/FISH.

In conclusion, we have shown excellent concordance between STRAT4 and IHC for ER and HER2 biomarker evaluation in tissues fixed and processed in a low-resource setting. Despite undocumented CIT and fixation time in our cohort, as well as very small tumor areas tested in a third of cases, results were obtained in 97% of cases tested. The STRAT4 cartridge for use on the GeneXpert Platform is an excellent innovative solution for the challenge of providing high-quality breast cancer biomarker data in pathology laboratories that do not have access to immunohistochemistry or that have insufficient case volumes to justify maintaining all reagents and engaging in quality assurance testing for breast cancer biomarkers.
